# Mitochondrial Function Under Combined Oral Contraceptive Exposure in a Neuroblastoma Model: A Preliminary Investigation

**DOI:** 10.3390/biomedicines14051064

**Published:** 2026-05-07

**Authors:** Francesco Chiara, Sarah Allegra, Francesco Maximillian Anthony Shelton Agar, Giuliana Abbadessa, Silvia De Francia

**Affiliations:** 1 Department of Physics, University of Trento, Povo, 38122 Trento, Italy; francesco.chiara@unito.it; 2 Department of Clinical and Biological Sciences, University of Turin, 10043 Orbassano, Italy; francescomaximilliananthony.sheltonagar@unito.it (F.M.A.S.A.); giuliana.abbadessa@unito.it (G.A.); silvia.defrancia@unito.it (S.D.F.)

**Keywords:** SH-SY5Y, mitochondrial membrane potential, JC-1, H_2_DCFDA/DCF, estradiol, progesterone, dienogest, ethinylestradiol, combined oral contraceptives, neuroendocrinology, oxidative stress, mitochondrial dynamics

## Abstract

**Background:** Endogenous estradiol/progesterone (E2/P4) regulates neuronal mitochondrial bioenergetics and redox balance. However, the effects of combined oral contraceptive (COC) steroids under physiologically relevant hormonal conditions remain poorly understood. Therefore, this study aims to investigate how COC steroids modulate mitochondrial function within the defined E2/P4 hormonal milieus. **Methods:** Human SH-SY5Y cells (Cytion) were starved for 48 h in Cytion medium without FBS and with 1% ITS, and then exposed for 48 h to six conditions: vehicle (F0); follicular-like E2/P4 (F1); luteal-like E2/P4 (F2); F1 + dienogest/ethinylestradiol (DNG/EE; F3); F2 + DNG/EE (F4); and DNG/EE alone (F5). The primary endpoints were mitochondrial membrane potential (JC-1 red/green ratio) and ROS (H_2_DCFDA/DCF); nitric oxide (DAF-FM) was also recorded. Hoechst 33342 nuclear fluorescence served both as a per-well proxy of cell number and as a proportionality factor for normalization. **Results:** Follicular- and luteal-like backgrounds were associated with distinct ΔΨm/ROS set-points. The addition of DNG/EE was accompanied by background-dependent shifts, generally characterized by higher DCF signals and lower JC-1 ratios relative to the vehicle, whereas DAF-FM did not reveal statistically robust changes in NO. Hoechst-based normalization preserved these patterns, suggesting that the observed effects likely reflect per-cell functional modulation rather than differences in cell number, although modest, background-dependent variations in proliferation were observed, with DNG/EE associated with a greater increase in ROS under follicular-like conditions and a more pronounced ΔΨm membrane potential in a luteal-like milieu. **Conclusions:** These findings suggest that neuroendocrine background may influence mitochondrial ΔΨm/ROS states in SH-SY5Y cells, with physiologically inspired E2/P4 milieus potentially shaping baseline conditions onto which COC components exert context-dependent effects at 48 h. These preliminary findings provide a standardized framework for subsequent image-based analyses of mitochondrial function.

## 1. Introduction

Mitochondria are a principal interface between steroid signaling and neuronal bioenergetics/redox control. Endogenous estrogens modulate respiratory chain efficiency, antioxidant defenses, and mitochondrial membrane potential (ΔΨm) through both nuclear (ERα/ERβ) and extranuclear signaling, including rapid actions that converge on mitochondrial proteins and biogenesis programs; in healthy neural contexts, these effects tend to favor resilience and efficient energy use, the “healthy-cell bias” of estrogenic action [1,2]. In parallel, progesterone and synthetic progestogens regulate mitochondrial function, calcium handling, and pro-survival pathways via classical and membrane-associated receptors, supporting neurotrophic and neuroprotective outcomes across diverse CNS models [3,4].

The steroid hormones are made from cholesterol and control immunity, metabolism, stress reactions, and reproduction [5]. Throughout the menstrual cycle, as well as throughout the lifespan, their levels vary in accordance with circadian and ultradian rhythms [6,7,8,9]. Steroid hormones communicate via nuclear receptors of the NR3 class. In order to bind co-regulatory proteins and move them to the nucleus, where they control gene transcription, the receptors go through conformational changes [10]. Furthermore, certain steroid hormone receptors, such as estrogen receptors, have the ability to indirectly control gene expression at certain promoters [11]. Additionally, the steroid hormones signal through non-genomic mechanisms that are initiated at the cell membrane by G-protein-coupled estrogen receptor 1 (GPER1) or palmitoylated steroid hormone receptor variants. These receptors activate kinase pathways and modulate gene expression by controlling the activity of growth factor receptors and cytoplasmic nuclear receptors [12].

The functions of steroid hormones in controlling immune cell growth, activation, and function, as well as intracellular metabolic pathways, have been discussed in a number of publications [13,14,15].

Against this backdrop, combined oral contraceptives (COCs)—typically ethinylestradiol (EE) plus a progestin (e.g., dienogest (DNG))—introduce exogenous steroids that can reshape systemic redox balance and inflammatory tone. Multiple human studies report alterations in oxidative stress biomarkers among COC users relative to non-users (e.g., hydroperoxides, antioxidant capacity, and enzyme activities), though the direction and magnitude vary with formulation, dose, and physiology, highlighting the need for controlled cellular models to resolve mechanism and context [16,17,18]. In neuronal-like systems, where mitochondrial function is tightly coupled to viability and signaling, parsing how exogenous EE/progestins interact with physiologic hormonal milieus is particularly germane.

The SH-SY5Y human neuroblastoma line is a standard neuronal-like model to assay mitochondrial and oxidative endpoints, suitable for systematic interrogation of steroid effects under defined conditions [19,20]. For functional readouts, JC-1 yields a radiometric red/green signal proportional to ΔΨm, whereas H_2_DCFDA/DCF provides a generic index of intracellular ROS; both are widely adopted when implemented with appropriate controls and awareness of probe caveats (e.g., dye loading/efflux, oxidation chemistry, and the necessity of orthogonal validation) [21,22,23]. Although widely used, DCF fluorescence reflects general intracellular oxidative activity and does not allow discrimination among specific reactive oxygen species, such as superoxide, hydrogen peroxide, or hydroxyl radicals [24]. Therefore, the changes should be interpreted as an overall redox imbalance rather than the production of a specific ROS. Similarly, JC-1 is a well-established indicator of mitochondrial membrane potential; however, its accumulation within mitochondria can be influenced by multidrug resistance (MDR) transporters, potentially affecting dye retention independently of mitochondrial polarization [25]. Since steroid hormones have been reported to modulate the expression and activity of efflux transporters, this factor should be considered when interpreting JC-1-based measurements. Beyond plate reader assays, Mitotracker imaging coupled with quantitative morphometry (e.g., area, aspect ratio, form factor, and perinuclear density) captures structural correlates of mitochondrial function, and QuPath provides an open, reproducible framework for high-throughput feature extraction and downstream statistics [26,27,28].

Physiological follicular and luteal milieus differ chiefly in progesterone (low vs. high), with estradiol spanning lower-to-mid-ranges across sub-phases; these fluctuations plausibly set distinct mitochondrial “set-points” in neural cells [29,30]. Emulating such backgrounds in vitro offers a principled way to test context-dependent actions of EE and progestins. Here, we use SH-SY5Y cells to (i) establish follicular-like and luteal-like conditions with physiologically inspired estradiol/progesterone (E2/P4) combinations; (ii) overlay DNG/EE on each background and as a standalone exposure; and (iii) quantify ΔΨm (JC-1) and ROS (DCF) after 48 h, a window chosen to capture stabilized, transcriptionally integrated bioenergetic responses beyond acute dye or ion-flux artifacts. Within this framework, our expectation is that the E2/P4 milieu defines baseline ΔΨm/ROS states and that DNG/EE imposes background-dependent shifts at 48 h, directionality that we will subsequently connect to Mitotracker-based morphometry (via QuPath) to bridge functional readouts with structural phenotypes. Overall, the objective of this study is to determine how COC steroids modulate mitochondrial function in a hormone-dependent context and to integrate functional and structural readouts of mitochondrial dynamics.

Our findings highlight the importance of considering the hormonal milieu—defined here as the specific combination and concentration of steroid hormones—as a key determinant of condition-dependent mitochondrial functional states (‘set-points’), reinforcing the need for physiologically relevant experimental designs when interpreting mitochondrial responses.

## 2. Materials and Methods

### 2.1. Reagents and Solutions

P4 (HY-N0437), E2 (HY-B0141), DNG (HY-B0084), and EE (HY-B0216) were standard-grade and purchased from MedChemExpress (Monmouth Junction, NJ, USA); stocks were prepared in DMSO and diluted in a medium to reach the final concentration indicated in Table 1 (final DMSO ≤ 0.1% *v*/*v*). All experimental conditions were balanced for the final DMSO concentration (≤0.1% *v*/*v*). The probes JC-1 (HY-K0601-100T), H_2_DCFDA (HY-153006), DAF-FM DA (HY-D0717), and Hoechst 33342 (HY-15559) were also purchased from MedChemExpress (Monmouth Junction, NJ, USA).

### 2.2. Data Quality, Replicates, and Normalization

Across plates, each condition (F0–F5) included *n* = 8 technical replicates. After the pre-registered robust QC/outlier handling, effective replicate numbers per condition remained adequate for non-parametric inference (median n_clean ≈ 5; range 5–8). Hoechst 33342-integrated fluorescence at 48 h was used both as a proxy of cell number/proliferation and as a proportionality factor for functional readouts (DCF; JC-1 ratio). Normalization to Hoechst did not invert qualitative patterns observed in raw signals.

### 2.3. Proliferation (Hoechst 33342)

Hoechst fluorescence indicated modest, background-dependent differences in cell number at 48 h. Relative to the vehicle (F0), follicular-like (F1) and luteal-like (F2) milieus showed only small changes in the Hoechst signal; adding DNG/EE (F3, F4) or using DNG/EE alone (F5) did not produce large proliferation shifts. Consequently, Hoechst-normalized endpoints closely tracked raw effects, suggesting per-cell functional modulation rather than artifacts from varying cell numbers.

### 2.4. Cell Line and Culture Conditions

Cells: SH-SY5Y human neuroblastoma cells (Cytion; Heidelberg, Germania) were maintained according to the Cytion technical datasheet.

Incubation: The incubation conditions were 37 °C, 5% CO_2_, and saturated humidity.

Starvation prior to treatment: The cells were starved for 48 h in Cytion medium without FBS, supplemented with 1% ITS (insulin–transferrin–selenium), to minimize exogenous steroid interference and synchronize metabolic state. The cells were then exposed to test conditions for 48 h.

### 2.5. Experimental Design and Treatment Conditions

Six conditions were tested (F0–F5), emulating follicular-like and luteal-like hormonal milieus, with or without combined oral contraceptive (COC) steroids. We thank the reviewer for raising this important point. The concentration of DNG used in our experiments (160 nM) was selected to reflect clinically relevant exposure levels. Specifically, 160 nM corresponds to approximately 47 ng/mL of dienogest, which falls within the reported range of peak serum concentrations (Cmax) in humans (approximately 20–50 ng/mL) [24].

The final DMSO was ≤ 0.1% *v*/*v* in all wells.

### 2.6. Plate Layout and Controls

The experiments were run in multi-well plates (96-well plates unless otherwise noted) following the “Piastre” map in the spreadsheet. Each condition included *n* = 8 technical replicates per plate. The controls included the following:

- Vehicle (F0; “CTR”), on every plate.
- Positive ROS control: H_2_O_2_ (100 μM) for 60 min (plate-end spike) where indicated.
- Antioxidant control: N-acetyl-L-cysteine (NAC) (0.5 mM), maintained throughout the 48 h exposure (where indicated).
- ΔΨm membrane potential control (for JC-1): CCCP/FCCP (50 μM) for 5 min before reading; plates kept protected from light.

### 2.7. Fluorimetric Assays

All probes were handled under low light; identical exposure/gain settings were used across conditions on the same plate.

### 2.8. JC-1 (Mitochondrial Membrane Potential (ΔΨm))

Cells were washed with PBS and incubated with JC-1 at the manufacturer-recommended working concentration for 20–30 min at 37 °C. Fluorescence was read at

- Green (monomer): Ex 485–490 nm/Em ~530 nm;
- Red (aggregate): Ex 525–540 nm/Em 590–610 nm;
- The red/green ratio was used as the primary ΔΨm endpoint.

### 2.9. H_2_DCFDA/DCF (ROS)

Cells were incubated with H_2_DCFDA (typically 10–20 μM) for 30 min at 37 °C, washed, and read at Ex 485–495 nm/Em 525–535 nm. Where plate-end H_2_O_2_ spikes were used, readings were taken immediately after the 60 min incubation.

### 2.10. DAF-FM Diacetate (Nitric Oxide (NO))

After 48 h treatment, the wells were washed and incubated with DAF-FM DA for 30–45 min at 37 °C, followed by a de-esterification period in probe-free buffer (15–30 min). Fluorescence was read at Ex ~495 nm/Em ~515 nm.

### 2.11. Hoechst 33342 (Nuclear Content/Proportionality)

At the end of the 48 h exposure, the nuclei were stained with Hoechst 33342 (0.5–5 μg/mL) for 10–15 min at 37 °C and read at Ex ~350–360 nm/Em ~460–470 nm. The integrated Hoechst signal per well was used both as a proxy for cell number/proliferation at 48 h and as a proportionality factor for normalization of functional readouts (DCF, JC-1 ratio). Hoechst normalization was used as a pragmatic correction for gross cell number differences, not as a biologically rigorous scaling factor for mitochondrial function.

### 2.12. Data Processing and Normalization

Raw plate data were exported to MATLAB (R2026a, The MathWorks, Natick, MA, USA) for analysis. For each well,

- Primary endpoints: JC-1 red/green ratio (ΔΨm), DCF (ROS), DAF-FM (NO), and Hoechst (nuclear signal).
- Normalization: In addition to raw values, we report Hoechst-normalized endpoints to correct for cell number bias:
  $${\mathrm{Endpoint}}_{\mathrm{norm}}=\frac{\mathrm{Endpoint}\;(\mathrm{DCF}\;\mathrm{or}\;\mathrm{JC}\text{-}1\;\mathrm{ratio})}{\mathrm{Hoechst}\;\mathrm{integrated}\;\mathrm{fluorescence}}$$
- Aggregation: Technical replicates (*n* = 8) were summarized per condition/plate before inferential testing.

### 2.13. Quality Control and Statistics

The data underwent visual QC and robust outlier handling (pre-specified rules). Unless distributions justified parametric tests, we used non-parametric approaches:

- Across-condition comparisons: Kruskal–Wallis.
- Overall differences among conditions were assessed using Welch’s one-way ANOVA, followed by Games–Howell post hoc pairwise comparisons. Selected planned contrasts, including F1–F5 vs. F0, F1 vs. F2, and background effect comparisons, such as F3 vs. F1 and F4 vs. F2, were also evaluated using Mann–Whitney tests with Benjamini–Hochberg FDR control (q = 0.05).
- Effect sizes: Median differences and rank-biserial correlation; 95% CIs by bootstrap where applicable.
- All analyses were run in MATLAB with custom scripts.

### 2.14. Statistical Analysis

All statistical analyses were performed using an approach designed to account for unequal variances among experimental groups and potential deviations from normality. Each experimental condition initially included *n* = 8 technical replicates per plate. Following quality control and outlier handling, the effective number of replicates per condition ranged from five to eight. Group-wise differences across experimental conditions were first assessed using Welch’s one-way ANOVA, which does not assume homogeneity of variances. When the global test indicated significant differences among groups, pairwise comparisons versus the control condition or between relevant experimental conditions were performed using Mann–Whitney U tests. To control for multiple testing, pairwise *p*-values were adjusted using the Benjamini–Hochberg false discovery rate procedure. Statistical significance was defined as FDR-adjusted *p* < 0.05. The data are presented as individual data points overlaid on box-and-whisker plots, with boxes representing the interquartile range and the central line indicating the median.

## 3. Results

### 3.1. ROS (DCF)

**Figure 1:**
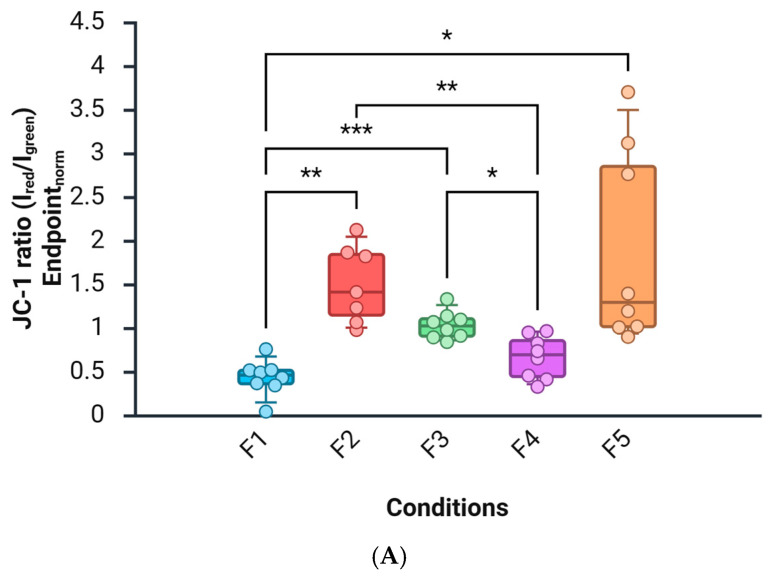

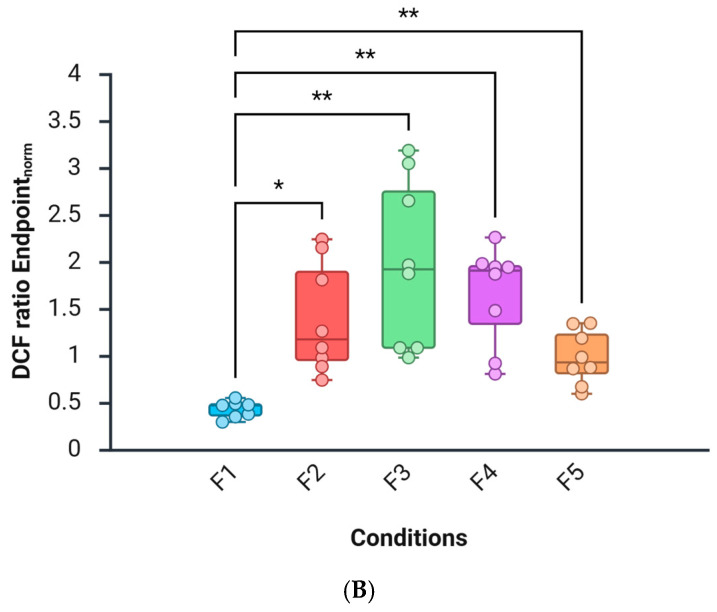
Distribution of DCF fluorescence (**B**) and JC-1 ratio (**A**) across experimental conditions. Part (**A**)—Box-and-whisker plot showing the JC-1 fluorescence ratio, expressed as $I_{red}/I_{green}$ and normalized to the endpoint norm (see Section 2.12), across conditions F1–F5. Individual data points are overlaid on each boxplot. Statistical analysis was performed using Welch’s one-way ANOVA, which revealed a significant difference between groups, where W(4,16)=15.610, and p<0.001. Post hoc pairwise comparisons are indicated by brackets. Asterisks denote statistical significance: * p<0.05, ** p<0.01, and *** p<0.001. Part (**B**)—Box-and-whisker plot showing DCF fluorescence, expressed as endpoint norm, across conditions F1–F5. Individual data points are overlaid on each boxplot. Statistical analysis was performed using Welch’s one-way ANOVA, which revealed a significant difference between groups, where W(4,15)=22.380, and p<0.001. Pairwise post hoc comparisons are indicated by brackets. Asterisks denote statistical significance: * p<0.05, ** p<0.01. Experimental groups are defined as follows: F0, vehicle control; F1, follicular-like background (E2/P4); F2, luteal-like background (E2/P4); F3, follicular-like background plus DNG/EE; F4, luteal-like background plus DNG/EE; and F5, DNG/EE exposure alone. For a detailed description of the hormonal concentrations used in each condition, please refer to Table 1.

The DCF differed significantly across conditions (Kruskal–Wallis, FDR-controlled). Versus F0, F1–F5 showed a higher DCF, with the largest increase in F3 (F1+ DNG/EE) and a marked elevation also in F4 (F2+ DNG/EE). F5 (DNG/EE alone) was elevated vs. F0 but lower than phase-plus-COC conditions. These contrasts remained significant after Hoechst normalization (pairwise Mann–Whitney, FDR; Figure 1B).

### 3.2. Mitochondrial Membrane Potential (JC-1 Ratio)

The JC-1 red/green ratio showed a significant across-group effect (Kruskal–Wallis, FDR). Relative to F0, F1–F5 exhibited a decrease in the JC-1 ratio (ΔΨm membrane potential), most pronounced in F4 (luteal-like + DNG/EE) and substantial in F3 (follicular-like + DNG/EE). F5 showed a smaller but consistent decrease. Effects persisted after Hoechst normalization (pairwise Mann–Whitney, FDR; Figure 1A). To further characterize the impact of the hormonal background, we directly compared the two control conditions, F1 (follicular-like) and F2 (luteal-like). While no significant difference was observed in basal ROS levels between F1 and F2 (*p* > 0.05; Mann–Whitney test), the mitochondrial membrane potential (JC-1 ratio) showed a distinct baseline, with F2 maintaining a higher ratio compared with F1 (*p* < 0.05). These findings support the hypothesis that the two hormonal milieus establish distinct bioenergetic ‘set-points’ before COC exposure.

### 3.3. Nitric Oxide (DAF-FM)

DAF-FM did not differ significantly across conditions (Kruskal–Wallis, n.s.), and no pairwise comparison vs. F0 survived FDR correction. Thus, within 48 h, NO changes were not robust in this model under the tested exposures. Overall, our results consistently indicate that steroid treatments modulate key physiological outputs, including oxidative status, across different neuroblastoma phenotypes, supporting a context-dependent mitochondrial response.

### 3.4. Background Dependence (Follicular vs. Luteal Milieus)

**Figure 2:**
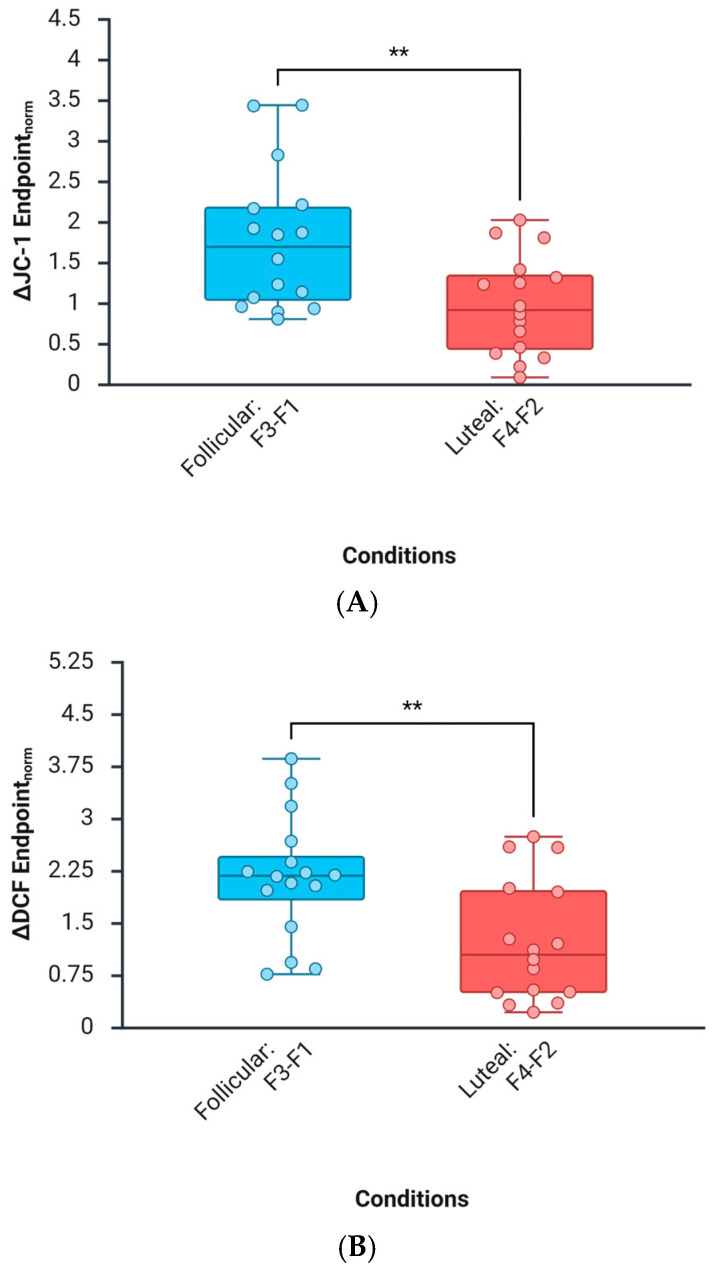
Box-and-whisker plots showing ΔDCF fluorescence (**B**) and ΔJC-1 (**A**) ratio, expressed as endpoint norm, in the follicular condition F0–F1 and the luteal condition F0–F2. Individual data points are overlaid on each boxplot. For ΔDCF fluorescence, statistical analysis was performed using regular one-way ANOVA, which revealed a significant difference between groups, where F(1,30)=8.789, and p=0.006. For the ΔJC-1 ratio, regular one-way ANOVA also revealed a significant difference between groups, where F(1,30)=8.848, and p=0.006. Asterisks indicate statistical significance: ** p<0.01.

Comparisons of F3 vs. F1 and F4 vs. F2 support a background-dependent modulation by DNG/EE:

- ROS (DCF): The largest ROS rise occurred on the follicular-like background (F3 > F4).
- ΔΨm (JC-1): The strongest mitochondrial membrane potential occurred on the luteal-like background (F4 > F3).
- A simple interaction contrast (F4–F2) vs. (F3–F1) was directional for both endpoints, indicating that phase-like hormonal context primes mitochondrial responses to COC steroids at 48 h (Figure 2A,B).

### 3.5. Sensitivity Analyses and Controls

Sensitivity analyses (with/without Hoechst normalization; with/without aggressive outlier trimming) did not change the direction of main effects. Where included, positive/negative controls behaved as expected (e.g., H_2_O_2_ increased DCF; CCCP/FCCP decreased the JC-1 ratio), supporting assay validity.

## 4. Discussion

Steroid hormones are known to modulate intracellular redox balance, with several studies reporting changes in ROS production following hormonal exposure. E2, in particular, has been shown to exert context-dependent effects, acting as an antioxidant under certain conditions through upregulation of mitochondrial efficiency and antioxidant defenses, while also promoting ROS generation via estrogen receptor-mediated signaling and mitochondrial activity in other settings [31,32]. P4 similarly influences mitochondrial function and oxidative status, although its effects appear more variable and dependent on cell type and metabolic context [33]. Synthetic steroids used in COCs, including EE and progestins such as DNG [31], have also been associated with modulation of oxidative stress pathways, potentially through interactions with mitochondrial respiration and redox-sensitive signaling cascades [34]. Overall, these findings support the concept that hormonal exposure can dynamically regulate ROS production, with outcomes that depend on hormonal composition, concentration, and cellular context.

This preliminary study underscores the importance of the neuro-endocrine background in shaping mitochondrial readouts in neuronal-like cells. Using SH-SY5Y cells exposed for 48 h to phase-inspired E2/P4 milieus with or without dienogest/ethinylestradiol (DNG/EE), we observed two consistent functional outcomes: (i) a significant rise in DCF (ROS) and (ii) a decrease in the JC-1 red/green ratio (ΔΨm membrane potential) relative to the vehicle. These effects persisted even after Hoechst proportionality correction, indicating that changes reflect per-cell modulation rather than differences in cell number/proliferation at 48 h. Specifically, we hypothesize that high P4 levels in the luteal-like state may modulate the cell’s redox capacity by altering the expression or enzymatic activity of antioxidant defenses, such as superoxide dismutase (SOD) or catalase. A higher ‘antioxidant shield’ in the F2 background could explain why DNG/EE exposure leads to significant ΔΨm shifts without the massive ROS surge observed in the follicular-like (F1) background. Moreover, the pharmacological profile of Dienogest as a progestin suggests potential competitive or synergistic interactions with endogenous P4 at the level of nuclear (PGR) or mitochondrial progesterone receptors. In the high-P4 luteal background, the occupancy of these receptors might favor pathways that prioritize mitochondrial membrane stabilization or remodeling over direct oxidative damage. In contrast, no significant differences emerged for DAF-FM (NO) following multiple-testing correction. The absence of NO changes can be interpreted in light of the fast, transient dynamics of nitric oxide and the well-documented methodological limitations of DAF probes (e.g., oxidant cross-reactivity and esterase dependence), which reduce sensitivity to subtle condition-specific fluctuations [35,36,37].

The hormonal phase-like backgrounds modulated the magnitude and the direction of the responses. Adding DNG/EE on a follicular-like milieu (F3) produced the strongest ROS increase, whereas the luteal-like milieu with DNG/EE (F4) showed the most pronounced mitochondrial depolarization. These observations suggest that the E2/P4 set-point primes mitochondrial susceptibility, a concept consistent with growing evidence that sex steroids regulate mitochondrial bioenergetics, redox homeostasis, and vulnerability to metabolic stress [38,39,40,41,42,43,44,45]. Lower P4 in the follicular-like context may permissively amplify ROS, while higher P4 in the luteal-like condition may interact with progestin/estrogen signaling to bias ΔΨm shifts [46,47]. Although receptor engagement was not directly measured, the background-dependent outcomes align with modern models of steroid signaling that include ER/PR/GPER/mPR and direct mitochondrial receptor targets [38,48]. The dynamic role of sex hormones in neuronal mitochondrial function—including regulation of OXPHOS, antioxidant systems, and biogenesis—supports this interpretation [42,43,44,45,49,50].

Our assay validation strengthens confidence in these findings: H_2_O_2_ increased DCF as expected, and CCCP/FCCP decreased the JC-1 ratio, confirming mitochondrial sensitivity. Importantly, Hoechst-based additional proportionality affirmed that functional effects were not artifacts of altered cell number.

**Figure 3:**
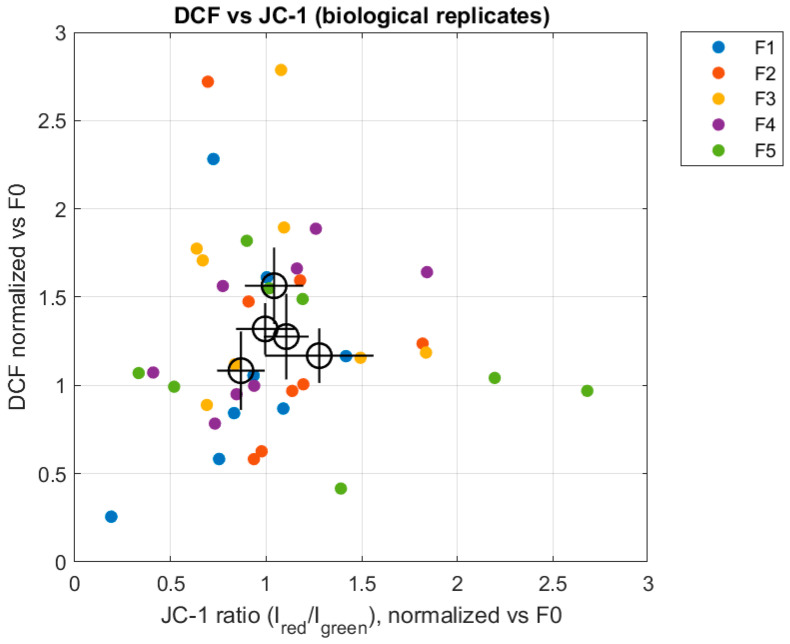
Relationship between mitochondrial polarization (JC-1 ratio) and oxidative stress (DCF fluorescence). Individual points represent biological replicates, colored by experimental condition. Larger open symbols indicate condition means ± SEM, highlighting condition-specific relationships between the two readouts.

The joint analysis of JC-1 and DCF signals further supports the notion that oxidative stress and mitochondrial polarization represent partially independent outputs of the cellular response to the hormonal environment. As shown in Figure 3, similar JC-1 ratios can be associated with different levels of DCF fluorescence depending on the experimental condition, suggesting that the progestinic index modulates redox balance beyond its effects on mitochondrial membrane potential.

Overall, our findings suggest that phase-inspired hormonal milieus (follicular vs. luteal) may be associated with differences in mitochondrial ROS and ΔΨm, with COC-like steroids inducing context-dependent changes at 48 h. This aligns with modern neuroendocrine-mitochondrial models positing that hormonal status is not a passive backdrop, but an active determinant of mitochondrial responsiveness [38,42,43,44,45,46]. This integrated view (hormones × mitochondria × receptor signaling) has implications for understanding how menstrual cycle phase, hormonal therapies, and contraceptives may modulate neuronal resilience, redox state, and susceptibility to metabolic or oxidative stress.

From a translational perspective, these findings raise relevant questions: Do fluctuating steroid levels during the menstrual cycle alter neuronal mitochondrial vulnerability? Could long-term exposure to combined oral contraceptives reshape neuronal mitochondrial baseline states? Such questions require validation in more physiological systems—including primary neurons, brain organoids, and in vivo models—as well as functional endpoints involving neuronal survival, synaptic signaling, and plasticity. Recent work underscores how sex hormones influence CNS energy metabolism and oxidative pathways [46,47], supporting the relevance of hormonal context in mitochondrial research.

Nevertheless, several limitations should be acknowledged. Although SH-SY5Y cells display neuron-like characteristics, they do not fully recapitulate the structural and functional complexity of mature neurons. In addition, our analysis was limited to a single exposure time point (48 h), which may reflect a stabilized, integrated response and fail to capture earlier, transient dynamics. Finally, the JC-1 and DCF assays present inherent methodological limitations, particularly regarding their specificity and quantitative accuracy, which should be considered when interpreting the results [51]. Although Hoechst normalization provides a pragmatic correction for cell number, it does not represent a biologically rigorous scaling factor for mitochondrial function. In particular, if treatments affect mitochondrial mass or volume per cell, normalization to cell number may obscure or distort treatment-related differences, and this limitation should be considered when interpreting the results. In addition, the observed variability reflects the intrinsic biological heterogeneity of the system. Future studies including a higher number of biological replicates will be important to further strengthen the robustness and generalizability of these findings. Likewise, we did not include orthogonal respiration or NO assays. These boundaries motivate the next experimental phase, which will incorporate Mitotracker-based morphometry (QuPath) to test structural correlates of ΔΨm/ROS trends, complemented—if desired—by orthogonal functional endpoints (e.g., TMRE/TMRM for ΔΨm, and MitoSOX/Amplex assays for ROS subtypes) and time-course sampling to capture early NO transients. Moreover, undifferentiated SH-SY5Y cells are cancer-derived, metabolically atypical, and do not recapitulate mature neuronal physiology. Our intention was not to model physiological neuroendocrine regulation in vivo, but to employ a reductionist, reproducible neuronal-like system to explore whether defined estradiol/progesterone backgrounds modulate mitochondrial and redox-sensitive readouts under controlled conditions.

## 5. Conclusions

In conclusion, our findings suggest that E2/P4 hormonal milieus may influence the mitochondrial state of SH-SY5Y cells, with follicular- and luteal-like backgrounds showing differences in ΔΨm and ROS at 48 h. Within these contexts, COC components appear to exert background-dependent effects, with DNG/EE associated with a greater increase in ROS under follicular-like conditions and a more pronounced ΔΨm in a luteal-like milieu. These outcomes reflect per-cell changes rather than differences in cell number, as supported by the preserved Hoechst proportionality of DCF/JC-1 signals and the minimal variation in proliferation. Conversely, NO levels were not significantly modulated at 48 h, as DAF-FM measurements did not reveal between-group differences after FDR correction. Finally, it should be considered that steroid-mediated modulation of MDR may influence JC-1 accumulation, and, thus, the observed changes in the JC-1 ratio may not exclusively reflect alterations in ΔΨm, but could also partially derive from differences in dye efflux.

However, these findings should be interpreted with caution, given the preliminary nature of this study and the use of a single in vitro neuronal-like model, which does not fully recapitulate the complexity of mature neuronal systems. While the present work provides a standardized framework for subsequent image-based analyses of mitochondrial function, further studies are needed to validate these observations using complementary models and orthogonal approaches. In particular, future investigations should incorporate real-time monitoring of NO and explore the temporal kinetics of ΔΨm and ROS changes, as the 48 h endpoint likely reflects a stabilized, integrated response and may overlook earlier, transient dynamics.

Taken together, our results point to a potential influence of the neuroendocrine context on the mitochondrial response to combined oral contraceptive steroids in neuronal-like cells; however, these observations require further confirmation before broader generalization.

## Figures and Tables

**Table 1:** Nominal compositions (per well).

Condition	Progesterone (P4) [nM]	Estradiol (E2) [nM]	Dienogest (DNG) [nM]	Ethinylestradiol (EE) [nM]	Description
**F0**	0	0	0	0	Vehicle (control)
**F1**	1.87	0.28	0	0	Follicular-like background
**F2**	40.0	0.50	0	0	Luteal-like background
**F3**	1.87	0.28	160	0.30	F1 + DNG/EE
**F4**	40.0	0.50	160	0.30	F2 + DNG/EE
**F5**	0	0	160	0.30	DNG/EE only

## Data Availability

The datasets generated during and/or analyzed during the current study are available from the corresponding author upon request.
